# [Corrigendum] Heat shock protein 22: A new direction for cardiovascular disease (Review)

**DOI:** 10.3892/mmr.2025.13535

**Published:** 2025-04-14

**Authors:** Yi Chen, Meng Li, Yanqing Wu

Mol Med Rep 31: 82, 2025; DOI: 10.3892/mmr.2025.13447

Subsequently to the publication of this paper, the authors were contacted by an interested reader who was concerned that they had reported some erroneous information regarding the structure and properties of small heat-shock protein Hsp22, and its participation in different aspects of cardiovascular diseases.

After having considered the reader's comments (with which the authors agree), they have proposed the following change should be made to the text of the paper: The opening sentence of the section “**2. Protective effect of HSP22 on myocardial cells**” in the left-hand column of p. 2, instead of reading “HSP22 is a transmembrane protein with a stable tertiary structure, which contains long α-helices and short β-curls that result in a high degree of stability (9,14).”, should be replaced by the following text: “**HSP22 has an α-crystallin domain next to the C-terminus and is mainly found in the cytoplasm** (9,14).

The authors regret that erroneous information that was included in the published review, and are grateful to the Editor of *Molecular Medicine Reports* for granting them the opportunity to publish this corrigendum. Furthermore, they thank the interested reader for drawing this matter to our attention, and apologize to the readership for any inconvenience caused.

